# A prospective observational study measuring the stability of plasma pTau181 using the Elecsys Phospho-Tau (181P) plasma immunoassay for early detection of Alzheimer’s disease

**DOI:** 10.1186/s13195-026-02238-7

**Published:** 2026-10-24

**Authors:** Carolin Kurz, Sophia Leonhardt, Anna Hufnagel, Sabine Wizemann, Heike Deutelmoser, Patrick Menzel, Robert Perneczky

**Affiliations:** 1 Department of Psychiatry and Psychotherapy, LMU University Hospital, LMU Medizin, Ludwig-Maximilians-Universität München, Munich, 80336 Germany; 2 Department of Psychiatry and Psychotherapy, School of Medicine and Health, Technical University Munich, Munich, 81675 Germany; 3 Study Delivery CVMD Core Lab, Roche Diagnostics GmbH, Penzberg, 82377 Germany; 4 Biostatistics & Data Science, Roche Diagnostics GmbH, Penzberg, 82377 Germany; 5 Assay Development New Products – PD PHCS, Roche Diagnostics GmbH, Penzberg, 82377 Germany; 6 German Center for Neurodegenerative Diseases (DZNE), Munich, 81377 Germany

**Keywords:** Alzheimer’s disease, Analyte recovery, Blood-based biomarkers, Diagnosis, Early detection, Immunoassay, pTau181, Sample stability

## Abstract

**Background:**

Plasma tau phosphorylated at threonine 181 (pTau181) is a key blood-based biomarker that correlates with the burden of amyloid-beta (Aβ) pathology in Alzheimer’s disease (AD). The Elecsys^®^ Phospho-Tau (181P) Plasma (pT181p) immunoassay (Roche Diagnostics International Ltd, Rotkreuz, Switzerland) is intended for use as an aid in the early detection of Aβ pathology in adults ≥ 55 years presenting with complaints of cognitive decline and is Conformité Européenne-marked and approved for clinical use by the US FDA. Here, we evaluated the stability of plasma pTau181 measured using the pT181p immunoassay under different pre-analytical and storage conditions in samples from an intended-use population.

**Methods:**

Whole blood samples from individuals presenting at LMU Hospital with symptom severity ranging from subjective cognitive decline to moderate AD dementia were collected in three different tubes for plasma preparation (Li-Heparin, K2-EDTA, and K3-EDTA). These were transported under cooled (never frozen) conditions to the measurement site and processed within 6 h of collection. Plasma samples were stored at different temperatures for varying durations, and impact on plasma pTau181 recoveries was assessed.

**Results:**

Out-of-specification results not meeting pre-defined acceptance criteria observed in an initial cohort (*n* = 14), were found to be the result of buffy coat/erythrocyte contamination. Pre-analytical procedures were modified to prevent contamination during post-centrifugation plasma transfer. In samples from a separate cohort (*n* = 10), no instabilities were observed and acceptance criteria were met. After considering safety margins, analyte stability for all sample types was determined to be 2 days at 25 °C, 7 days at 4 °C, 12 weeks at -20 °C, and 16 weeks at -80°C; stability was also observed after one freeze/thaw cycle.

**Conclusions:**

pT181p immunoassay measurements were stable and robust with respect to different sample handling procedures and storage conditions. These findings support implementation of the assay into routine, non-specialist healthcare settings to facilitate practical blood-based testing to rule out AD-related Aβ pathology.

**Supplementary Information:**

The online version contains supplementary material available at https://doi.org/10.1186/s13195-026-02238-7.

## Background

Alzheimer’s disease (AD) is a chronic neurodegenerative disorder that can progress from mild memory loss to dementia [1], and is characterized by the pathologic accumulation of extracellular amyloid-beta (Aβ) plaques and intracellular neurofibrillary tangles [2]. With the arrival of disease-modifying therapies, such as lecanemab and donanemab [3, 4], it has become clear that early diagnosis and intervention are key to managing this debilitating condition. These therapies aim to slow the progression of AD-associated Aβ pathology [5], and the identification of at-risk individuals can help delay or prevent the progression of dementia [6].

Confirmatory testing for Aβ pathology currently relies on the well-validated methods of amyloid-positron emission tomography or cerebrospinal fluid biomarker analysis to demonstrate the presence of amyloid deposition in the brain [7–9]; however, these modalities are associated with limited accessibility, high costs [10], and invasiveness [11]. Furthermore, the effective and timely detection of individuals with suspected Aβ pathology can be hindered by factors including a general lack of awareness and stigma around AD, complex clinical guidelines, limited resources and competing healthcare priorities, and slow referral pathways to, and limited availability of, dementia specialists [6, 12–14]. These barriers create a substantial bottleneck in healthcare systems for individuals with suspected AD accessing confirmatory testing, which is crucial for informing clinical decision-making and onward management.

Blood-based biomarkers (BBBMs) are emerging as minimally invasive [15] and scalable tools [16] that can be used to support risk stratification and likelihood assessments early in the AD diagnostic pathway. The 2024 Revised Criteria for Diagnosis and Staging of AD underscored the need for a biologic basis for diagnosing and staging AD rather than evaluation of clinical symptoms alone, and highlighted the importance of incorporating highly accurate BBBMs into AD diagnostic workup [17]. BBBM tests with performance characteristics suitable for early rule out of Aβ pathology offer particular value for reducing unnecessary and invasive assessments in individuals with a low likelihood of Aβ pathology, while prioritizing individuals not ruled out for further confirmatory testing for AD [18, 19]. Implementation of BBBM testing into frontline (non-specialist) healthcare settings offers a potential solution to address the current system bottleneck in the AD diagnostic pathway [15]. BBBMs are the ideal tools to be used in settings such as primary care, as they offer the advantage that individuals are not required to attend specialist centers for the purpose of sample collection. Ideally, healthcare professionals could collect samples in their local clinical practice without the need for complex storage requirements prior to subsequent sample processing and analysis and have confidence in the biomarker robustness and practicability of use in daily clinical routine.

Plasma tau phosphorylated at threonine 181 (pTau181) is a key BBBM that correlates with the burden of AD-related Aβ pathology, as measured by amyloid-positron emission tomography, and is associated with cognitive impairment assessed by standard clinical scales across early stages of the AD continuum [20–22]. The Elecsys^®^ Phospho-Tau (181P) Plasma (pT181p) immunoassay (Roche Diagnostics International Ltd, Rotkreuz, Switzerland) was developed as a rule-out test for Aβ pathology in adults aged ≥ 55 years presenting with signs, symptoms, or complaints of cognitive decline [23, 24]. It is the first BBBM test, with Conformité Européenne marking developed under the in vitro diagnostic regulation as a standalone triage tool, which can be used across all care settings, to rule out amyloid pathology related to AD, and the first plasma biomarker test cleared by the US Food and Drug Administration to rule out AD-related amyloid pathology [25, 26]. The test is intended to streamline the AD diagnosis and management pathway by supporting the identification of individuals with a low likelihood of AD-related Aβ pathology, who may be directed for further evaluation for alternative (non-AD) causes of their cognitive complaints, while those who are not ruled out can be routed onto confirmatory testing for suspected AD [23, 24].

Robust validation of the analytical performance, including sample stability, is essential to demonstrate the suitability and reliability of the pT181p immunoassay for its intended use and to support implementation of the assay into routine clinical practice [27]. Here, we report the stability of the plasma pTau181 protein analyte measured using the Elecsys pT181p immunoassay under different storage/pre-analytical conditions, using samples that were freshly collected from individuals enrolled from an intended-use population and were not frozen prior to baseline measurement at time point 0 ([baseline plasma pTau181 measurement] t_0_).

## Methods

### Study design and participants

This prospective, observational study investigated the stability of the plasma pTau181 protein analyte measured with the pT181p immunoassay using different plasma collection tubes and across a range of storage temperatures (25 °C, 4 °C, -20 °C, and − 80 °C) and durations (up to a maximum of 18 weeks) (Table 1). Whole blood samples were obtained from adults attending the Department of Psychiatry and Psychotherapy, LMU Hospital Munich, Germany, who were under evaluation for subjective cognitive decline, or who had a diagnosis of mild AD or moderate AD dementia. Key criteria for inclusion were age 40–89 years and no contraindications to donating 60 mL of whole blood (e.g., severe blood clotting disorders, anemia, acute inflammation at the injection site, or allergies to disinfectants or latex). Key exclusion criteria included pregnancy or lactation, recent use of any investigational treatment, and use of any anti-amyloid treatment. In total, 46 individuals who met the inclusion criteria were screened at the sample collection site, of whom 24 were subsequently enrolled in the study.

**Table 1:** Stability storage temperatures, testing time points, and allowed measurement time windows for plasma pTau181

Storage temperature (acceptable range), °C	Measurement time points from t_0_					Allowed measurement time windows
	t_1_	t_2_	t_3_	t_4_	t_5_	
25 (15 to 25)	9 h	24 h	40 h	48 h	72 h	For t_1_, t_2_, and t_3_: +1 h For t_4_ and t_5_: measurements must be performed on the scheduled day, keeping to the same time of the day as t_0_
4 (2 to 8)	2 days	4 days	6 days	7 days	8 days	Measurements must be performed on the scheduled day, keeping to the same time of the day as t_0_
-20 (-15 to -25)	3 weeks	6 weeks	9 weeks	12 weeks	13 weeks	Measurements must be performed on the scheduled week, keeping to the same day of the week as t_0_
-80 (-60 to -90)	1 month (4 weeks)	2 months (8 weeks)	3 months (12 weeks)	4 months (16 weeks)	4 months + 2 weeks (18 weeks)	Measurements must be performed on the scheduled week, keeping to the same day of the week as t_0_

*Abbreviations: pTau181* tau phosphorylated at threonine 181, *t*_*0*_ time point 0 (baseline plasma pTau181 measurement), *t*_*1–5*_ measurement time points 1–5

Prior to enrollment, potential participants were required to provide written informed consent. The study duration was limited to two visits at the collection site: one for provision of information and consent, and one for the blood draw. No follow-up visits were required. The study was conducted in full conformance with Good Clinical Practice guidelines, the ethical principles founded in the Declaration of Helsinki, and all applicable laws and regulations. Ethical approval was obtained from the ethics committee at LMU Hospital.

### Sample collection, processing, and measurement

**Fig. 1:**
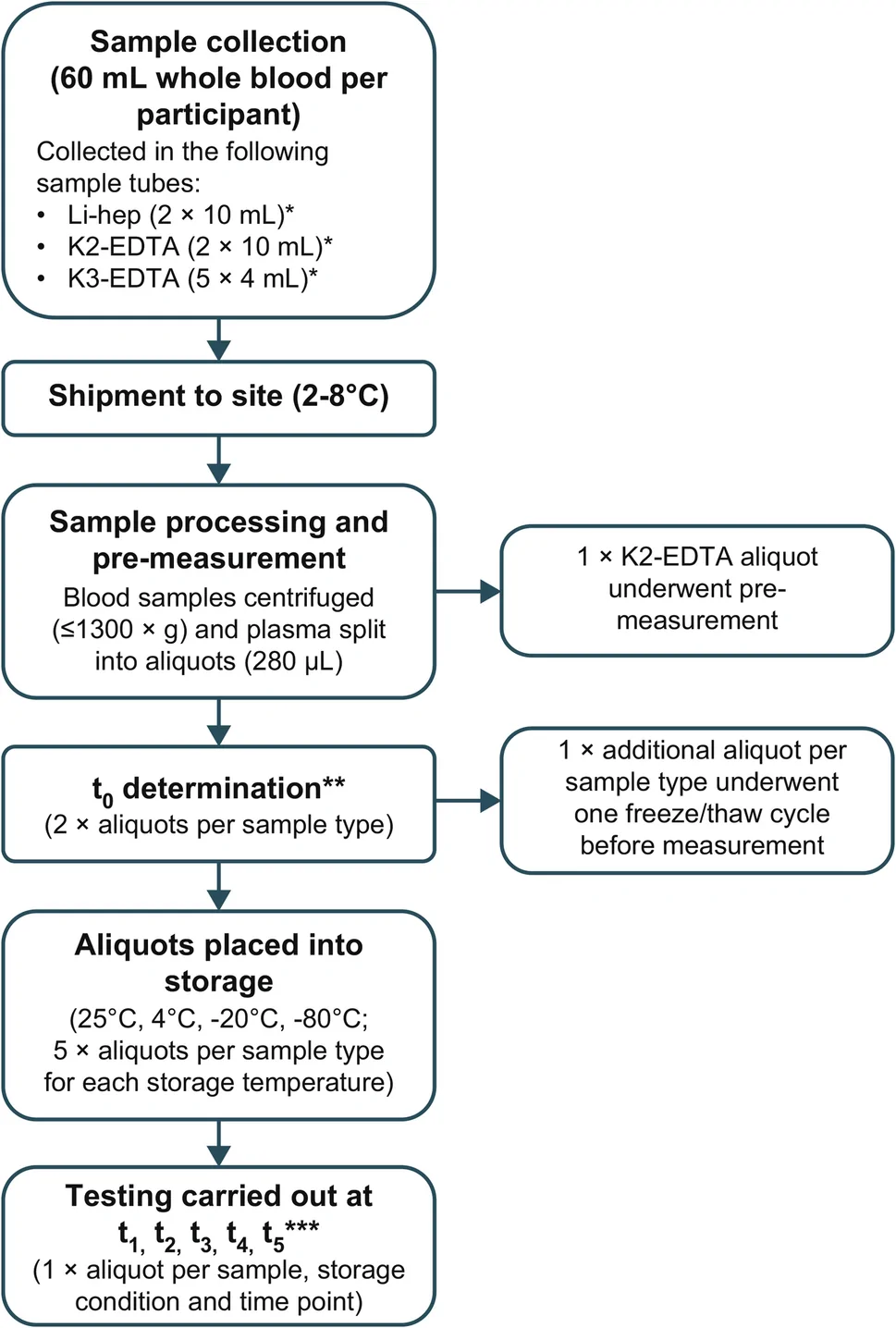
Sample collection and measurement workflow for the sample stability experiments. All samples were measured using the Elecsys pT181p immunoassay on a Cobas e 801 analyzer. *After study optimization, the sample collection tubes were changed to 6 × 4 mL. **Time taken from sample collection to t_0_ measurement did not exceed 6 h. ***See Table 1 for time points. Abbreviations K2-EDTA dipotassium ethylenediaminetetraacetic acid, K3-EDTA tripotassium ethylenediaminetetraacetic acid, Li-hep lithium-heparin, pT181p Phospho-Tau (181P) Plasma, t0 time point 0 (baseline plasma pTau181 measurement), t1–5 measurement time points 1–5

The workflow for sample collection and measurement is summarized in Fig. 1. A single blood draw was carried out to collect 60 mL per participant, using three different types of plasma collection tubes (all Becton Dickinson, Franklin Lakes, NJ, USA): 20 mL in lithium-heparin (Li-hep; 2 × 10 mL tubes); 20 mL in dipotassium ethylenediaminetetraacetic acid (K2-EDTA; 2 × 10 mL tubes); and 20 mL in tripotassium ethylenediaminetetraacetic acid (K3-EDTA; 5 × 4 mL tubes). This approach allowed assessment of whether sample type or distributed sample volumes affected analyte stability, thereby addressing practical variation encountered in real-world sample collection workflows. 

A tightly scheduled and monitored logistical process was followed for the 1–1.5 h transport duration from the collection site to the central measurement site. Whole blood samples were transported upright in dedicated tube racks under cooled (2–8 °C) but never frozen conditions in a thermally-insulated container monitored with temperature data loggers. Upon receipt at the central measurement site (TRIGA-S GmbH Scientific Solutions, Habach, Germany), samples were stored at 2–8 °C until processing.

To standardize pre-analytical processing, specimens were centrifuged at the testing site at ≤ 1300 × g for 10 min at room temperature in a swing-bucket centrifuge, with a mandatory minimum delay of 30 min post-collection to allow cell stabilization. The total time the whole blood samples remained uncentrifuged was short and did not exceed the threshold for cell degradation. Following centrifugation, plasma was immediately separated, pooled by sample type, and aliquoted into high-purity, low-protein binding 0.5 mL Sarstedt polypropylene microtubes (Sarstedt, Nümbrecht, Germany). The baseline (t_0_) measurement was completed for all samples within 6 h of the initial blood draw. Any specimen that failed to complete the baseline measurement within this 6-hour window was strictly excluded from the study to prevent pre-analytical bias. Plasma was then transferred into secondary tubes for each sample (280 µL per aliquot). Aliquots designated for stability testing were immediately placed into their respective temperature chambers. Low protein-binding tubes were used for aliquoting to prevent analyte loss through surface adsorption; 15 mL Sarstedt polypropylene tubes were used for native participant specimens that required pooling.

One aliquot of K2-EDTA plasma per participant was used to determine the preliminary sample concentration of plasma pTau181 using a Sarstedt microtube and performing a single measurement using the pT181p immunoassay on a Cobas^®^ e 801 analyzer (Roche Diagnostics International Ltd). This pre-measurement ensured that samples were within one of the specified concentration ranges, to avoid unnecessary measurements on invalid samples, and/or to provide details for excluding samples that were within concentration ranges that already had sufficient samples. This way, the entire assay measuring ranges were covered in the most efficient way. Concentration ranges were defined as low (plasma pTau181 ≥ 0.300 to ≤ 0.800 pg/mL), mid (> 0.800 to ≤ 2.00 pg/mL), and high (> 2.00 to ≤ 10.0 pg/mL).

All measurements were performed on a single e 801 analyzer and using the same Elecsys Phospho-Tau (181P) Plasma Reagent Kit (Lot 745749), CalSet Phospho-Tau (181P) Plasma Calibrator (Lot 745750), and PreciControl Phospho-Tau (181P) Plasma QC (Lot 745751) (all Roche Diagnostics International Ltd, Rotkreuz, Switzerland). Baseline plasma pTau181 measurements at t_0_ were taken using two aliquots of each sample type. After t_0_ measurement, a single aliquot per sample type was assigned to single freeze/thaw cycle (frozen at -20 °C overnight and left for a maximum of 3 days), and a further five aliquots per sample type were assigned to each of the following storage temperatures; 25 °C, 4 °C, -20 °C, and − 80 °C. Due to strict clinical, ethical, and pre-analytical constraints, only a single freeze-thaw cycle was performed. A single aliquot was used to measure plasma pTau181 analyte for the freeze/thaw cycle or storage temperature at various time points (Table 1). Before each measurement, aliquots were thawed, if necessary, homogenized, and measurement of plasma pTau181 was then conducted with the pT181p immunoassay on the e 801 analyzer, using channel assignment, with five replicates per aliquot.

To minimize the impact of analytical imprecision on stability modeling, baseline (t_0_) measurements were performed using two independent aliquots measured with five replicates each (*n* = 10 total baseline runs). Stressed storage aliquots (t_1–5_) were measured in five replicates (*n* = 5). Intra-aliquot precision was continuously monitored; the experimental coefficient of variation (CV) of the replicates consistently aligned with the assay’s verified within-run repeatability specifications (standard deviation ≤ 0.060 pg/mL for concentrations ≤ 1.00 pg/mL and CV ≤ 6.0% for concentrations > 1.00 pg/mL). Supplemental Tables 1 and 2 show a representative sample of raw analytical variability from a participant with a high concentration of plasma pTau181 (participant 50) and a participant with a low concentration of plasma pTau181 (participant 51), both stored at room temperature (25 °C) across all three sample types.

### Instrument calibration and quality control

Calibration of the e 801 analyzer was performed using CalSet Phospho-Tau (181P) Plasma Calibrator according to the description in the package insert provided to the measurement site [28]. Calibration was performed weekly (typically on a Tuesday before testing commenced), and once per reagent lot using a fresh reagent [28]. Every calibration result in combination with the corresponding quality control result were documented. There were specific target values for the acceptance criteria for the calibrations that were lot-dependent. The quality control (PreciControl Phospho-Tau [181P]) was run daily prior to any specimen measurements, and appropriate controls (PreciControl 1 and 2) had to be measured and ahead of every analytical run containing participant specimens to monitor the appropriate function of the instrument and validate the data within each run [28]. An analysis of the quality controls was performed prior to each run. Controls were handled according to the instructions stated in the manufacturer’s Instructions for Use. Operators verified that the controls were in the specified target range, and no Westgard rules were violated. The acceptance criteria for the quality controls were ± 18%.

### Statistical methods

It was determined that the study required ≥ 10 participant samples, based on an expected experimental coefficient of variation repeatability of the pT181p immunoassay of ≤ 4% and on an acceptable deviation of ± 10% recovery. Acceptance criteria for plasma pTau181 sample stability measurements were defined as follows: for samples with a t_0_ concentration ≤ 1.00 pg/mL, the allowable deviation was ≤ 0.100 pg/mL; for samples with a t_0_ concentration > 1.00 pg/mL, the allowable deviation was ≤ 10.0%. The final evaluation of sample stability was conducted using linear regression analysis across all recoveries. If the linear regression slope was not significantly different from 0 (*p* ≥ 0.05), the maximum storage time and sample shelf-life were considered equal to the maximum demonstrated time point. If the linear regression slope was significantly different from 0, the sample shelf-life was defined as the intercept between the confidence interval and acceptance criterion. The software used for analysis was R (version 4.4.1) [29].

While the pre-defined measurement windows ensured highly consistent operational workflows, any minor deviations outside of the planned hours did not impact the statistical validity of the study as the final stability evaluation was conducted via a linear regression model that utilized the actual (exact) time of measurement logged in WebCAEv, rather than the nominal planned time points, ensuring that minor temporal variations were mathematically accounted for and did not introduce analytical bias.

### Recovery calculation

The recovery percentage for each participant sample, time point, and storage condition was calculated as the mean stressed aliquot concentration for plasma pTau181, at any of the time points 1–5 under a designated storage condition (t_n_) divided by the mean concentration at baseline (t_0_). Recovery percentage = (mean concentration of stressed aliquots at t_n_) / (mean concentration of aliquots at t_0_) ⋅ 100%. This calculation was performed for every time point per participant, storage condition, and sample type.

### Optimization of study design

Initial results that did not meet the acceptance criteria were observed for several frozen Li-hep and K2-EDTA samples compared with the t_0_ value. The affected samples had been stored at.

-20 °C, -80 °C, or subjected to a freeze/thaw cycle. No samples stored at 25 °C, 4 °C, or any of the K3-EDTA samples were affected. Additional experiments were performed to investigate whether these out-of-specification results were due to sample instability or a sample handling issue, using the following conditions: K2-EDTA coated tubes, K2-EDTA gel separation tubes, K3-EDTA coated tubes; storage of the plasma collection tubes for 3 h at room temperature vs. 3 h at 4 °C; centrifugation directly after collection vs. after a 3-hour transport time at room temperature or 4 °C; sample centrifugation at 1300 × g or 1800 × g; storage at -20 °C and − 80 °C; and hemolytic vs. non-hemolytic samples.

As only hemolytic samples produced results that did not meet the acceptance criteria, issues with sample transportation were excluded as a cause, and hemolysis contamination effects were further evaluated. It was determined that reduced analyte recovery was observed when samples were contaminated with buffy coat and erythrocytes. Due to the large volume of plasma required to measure pTau181 at all experimental time points and replicates, it was found that all post-centrifugation plasma was being transferred to the secondary tube, leaving no safety buffer. However, the manufacturer of the plasma collection tubes recommends leaving a safety buffer of at least 0.5 mL of plasma, to prevent contamination from the buffy coat and erythrocytes. Thus, the sample collection procedures were updated. The 2 × 10 mL Li-hep tubes and the 2 × 10 mL K2-EDTA tubes were both replaced with 6 × 4 mL tubes. A sixth tube was also added to the K3-EDTA collection kit to ensure sufficient blood volume could be obtained and that plasma could be pipetted without contamination. Following the change in sample collection tube size, all previous samples were excluded, and blood draws from different individuals were used for the main evaluation of plasma pTau181 sample stability.

## Results

### Optimization of study design

The initial study involved 14 enrolled participants. The participants’ (who granted data-sharing consent [*n* = 7] and were included in this part of the study) were all White and not of Hispanic or Latino ethnicity (male, *n* = 5; female, *n* = 2). Three participants had a diagnosis of subjective cognitive decline and four had confirmed mild AD dementia. Participants’ age ranged from 69 to 82 years, with a mean age of 77.3 (standard deviation [SD] of 4.57) years.

Recoveries below an absolute deviation of 0.100 pg/mL for baseline ≤ 1.00 pg/mL or a relative deviation > 10% for baseline > 1.00 pg/mL relative to baseline, were observed for both K2-EDTA and Li-hep tubes. At -20 °C, 37 results were observed where values showed absolute deviations as low as -0.412 pg/mL for samples with baseline concentrations ≤ 1.00 pg/mL, and relative deviations down to -21.95% for samples with baseline concentrations > 1.00 pg/mL. At -80 °C, 19 results were observed where values showed absolute deviations down to -0.239 pg/mL for samples with baseline concentrations ≤ 1.00 pg/mL, and relative deviations down to -27.01% for samples with baseline concentrations > 1.00 pg/mL.

To confirm that reduced analyte recovery (in samples that were stored at -20 °C,

-80 °C, or were subjected to a freeze/thaw cycle) was due to buffy coat contamination, three samples were collected using the original 10 mL plasma collection tubes and analyzed specifically for feasibility. For two of the samples, a safety buffer was left in the tube. For one of the samples, the total volume of plasma was transferred to the secondary tube, with no plasma remaining to act as a safety buffer to prevent sample contamination. Under all conditions, the samples with a safety buffer showed no issues, whereas the sample without a safety buffer showed reduced analyte recovery after freezing.

### Sample stability results

Sample stability was determined by calculating recoveries of plasma pTau181 analyte compared with baseline measurements. Samples were obtained from 10 enrolled participants.

who were all White and not of Hispanic or Latino ethnicity (male, *n* = 5; female, *n* = 5). Five participants had a diagnosis of subjective cognitive decline and five had confirmed mild AD dementia. Participants’ age ranged from 69 to 86 years, with a mean age of 78.2 (SD of 5.073) years.

**Fig. 2:**
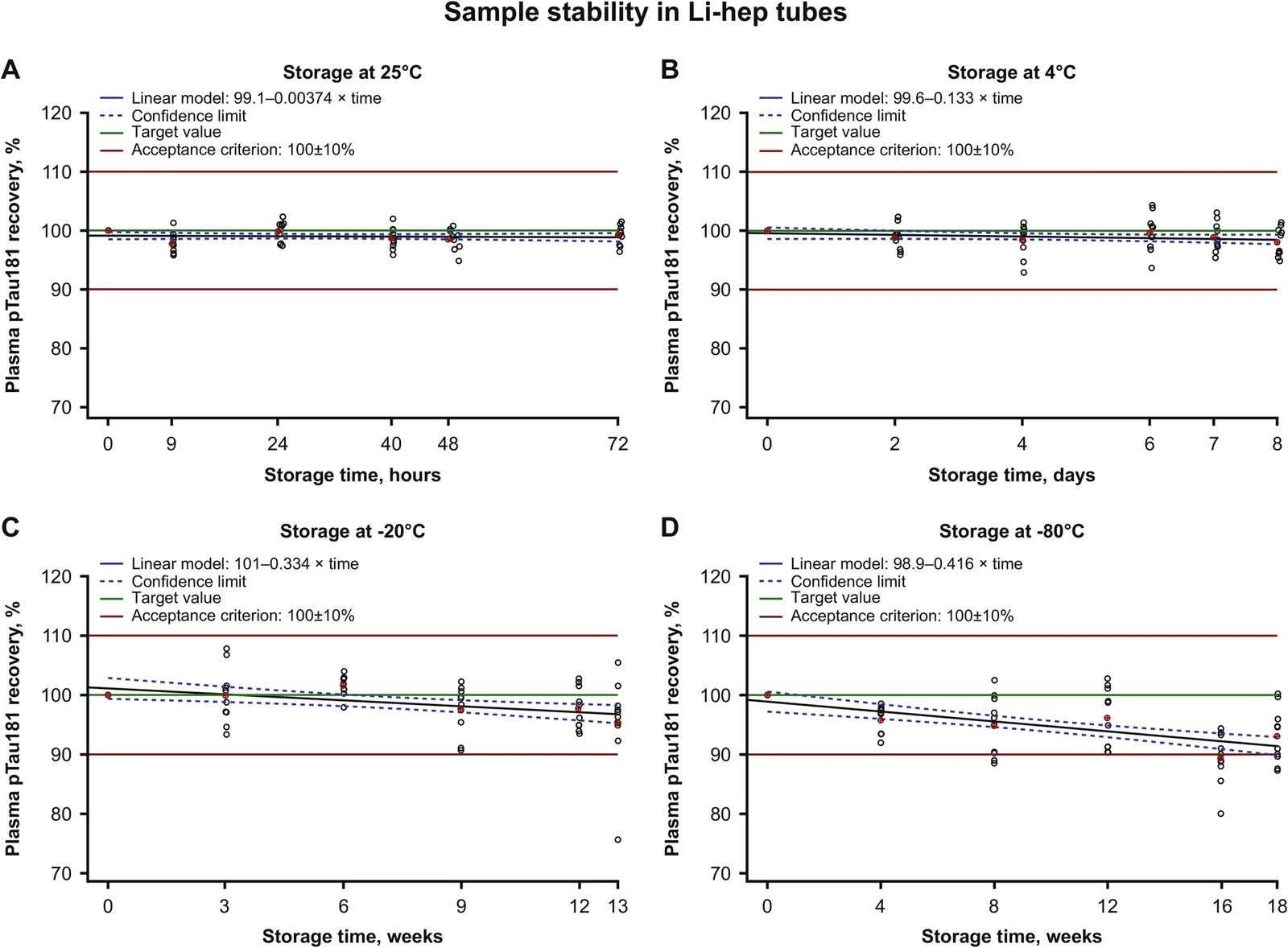
Scatter plot of plasma pTau181 stability regression in Li-hep tubes (25°C, 4°C, -20°C, and -80°C). Scatter plot of plasma pTau181 stability regression in Li-hep tubes stored at **A** 25°C for time points up to 72 hours, **B** 4°C for time points up to 8 days, **C** -20°C for time points up to 13 weeks, and **D** -80°C for time points up to 18 weeks. The solid blue line represents the fitted linear regression model, the dashed blue lines indicate the confidence limit, the solid green line represents the target value, and solid red lines indicate the acceptance criterion (100±10% recovery). Abbreviations Li-hep lithium-heparin, pTau181 tau phosphorylated at threonine 181

**Fig. 3:**
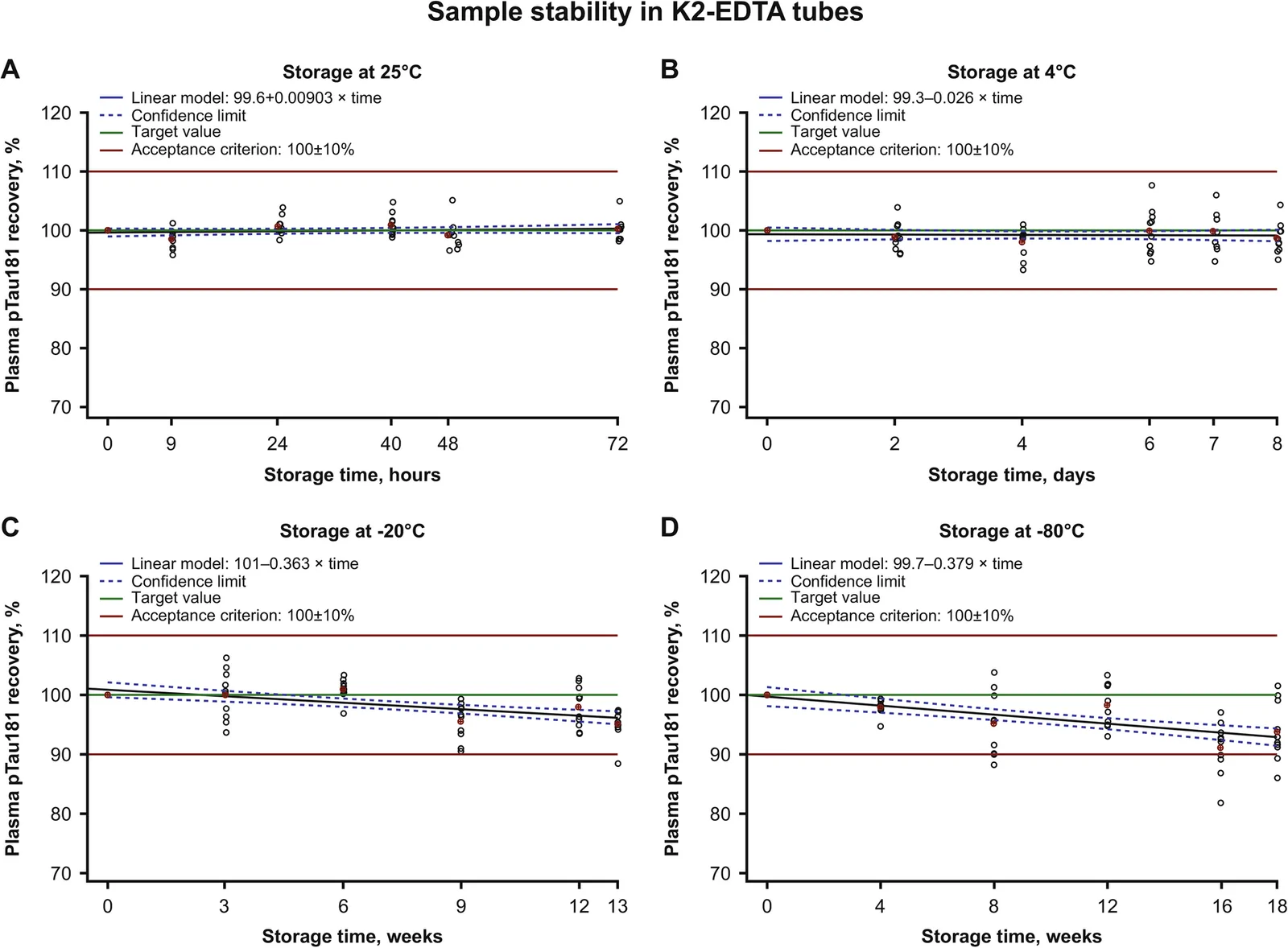
Scatter plot of plasma pTau181 stability regression in K2-EDTA tubes (25°C, 4°C, -20°C, and -80°C). Scatter plot of a linear regression model based on plasma pTau181 stability in K2-EDTA stored at **A** 25°C for time points up to 72 hours, **B** 4°C for time points up to 8 days, **C** -20°C for time points up to 13 weeks, and **D** -80°C for time points up to 18 weeks. The solid blue line represents the fitted linear regression model, the dashed blue lines indicate the confidence limit, the solid green line represents the target value, and solid red lines indicate the acceptance criterion (100±10% recovery). Abbreviations K2-EDTA dipotassium ethylenediaminetetraacetic acid, pTau181 tau phosphorylated at threonine 181

**Fig. 4:**
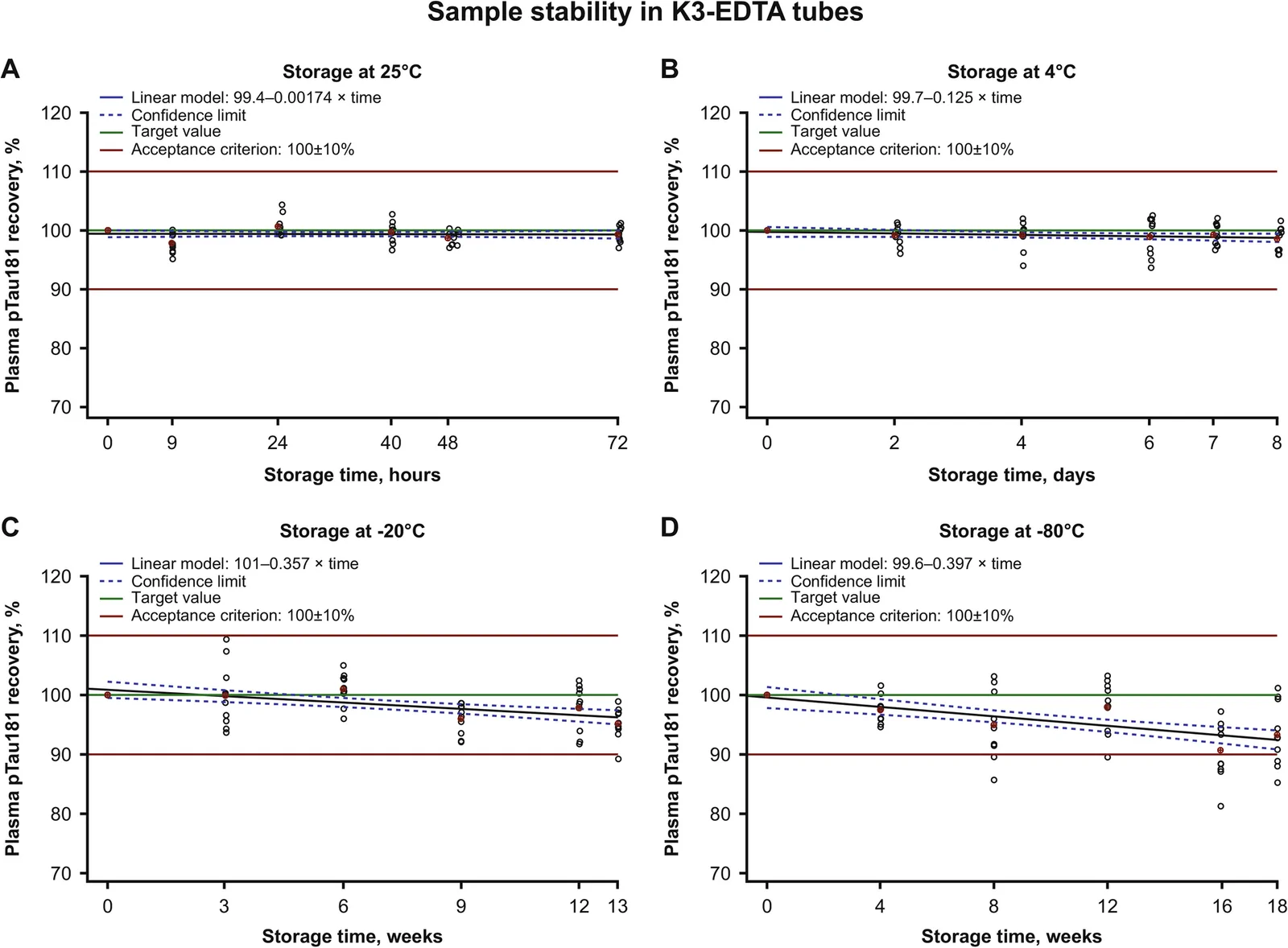
Scatter plot of plasma pTau181 stability regression in K3-EDTA tubes (25°C, 4°C, -20°C, and -80°C). Scatter plot of a linear regression model based on plasma pTau181 stability in K3-EDTA tubes stored at **A** 25°C for time points up to 72 hours, **B** 4°C for time points up to 8 days, **C** -20°C for time points up to 13 weeks, and **D** -80°C for time points up to 18 weeks. The solid blue line represents the fitted linear regression model, the dashed blue lines indicate the confidence limit, the solid green line represents the target value, and solid red lines indicate the acceptance criterion (100±10% recovery). Abbreviations K3-EDTA tripotassium ethylenediaminetetraacetic acid, pTau181 tau phosphorylated at threonine 181 -

Scatter plots of linear regression modeling based on plasma pTau181 recoveries across storage conditions for the Li-hep, K2-EDTA, and K3-EDTA tubes are shown in Figs. 2 and 3, and 4. After considering safety margins of 10–20% and regulatory guidelines, conservative plasma pTau181 analyte stability for all sample types was determined to be 2 days at 25 °C, 7 days at 4 °C, 12 weeks at -20 °C, and 16 weeks at -80 °C (Table 2).

**Table 2:** Maximum storage conditions by sample tube for samples measured using the Elecsys pT181p immunoassay

Sample tube	Storage temperature (°C)	Intercept (%)		Slope (%/unit)		*p*-value	Maximum storage time		
		PE	SE	PE	SE		Actual	Claimed	Unit
Li-hep	25 4 -20 -80	99.1 99.6 101 98.9	0.375 0.583 1.05 1.01	-0.00374 -0.133 -0.334 -0.416	0.00923 0.109 0.123 0.0870	0.6868 0.2292 0.0086 < 0.0001	72.0 8.00 13.0 17.8	65.0 7.00 12.0 16.0	hours days weeks weeks
K2-EDTA	25 4 -20 -80	99.6 99.3 101 99.7	0.403 0.685 0.748 0.962	0.00903 -0.0260 -0.363 -0.379	0.00992 0.129 0.0873 0.0830	0.3661 0.8413 0.0001 < 0.0001	72.0 8.00 13.0 18.0	65.0 7.00 12.0 16.0	hours days weeks weeks
K3-EDTA	25 4 -20 -80	99.4 99.7 101 99.6	0.357 0.502 0.818 1.06	-0.00174 -0.125 -0.357 -0.397	0.00879 0.0941 0.0955 0.0915	0.8438 0.1887 0.0004 < 0.0001	72.0 8.00 13.0 18.0	65.0 7.00 12.0 16.0	hours days weeks weeks

Maximum storage conditions based on linear regression estimates of recovery over time for the three different sample tubes, under four storage conditions. Data are based on *n* = 10 plasma samples per sample tube. All values were within the acceptance criterion of 100 ± 10% recovery

*Abbreviations: K2-EDTA* dipotassium ethylenediaminetetraacetic acid, *K3-EDTA* tripotassium ethylenediaminetetraacetic acid, *Li-hep* lithium-heparin, *PE* point estimate, *pT181p* Phospho-Tau (181P) Plasma, *SE* standard error

The absolute or relative deviations of plasma pTau181 analyte recoveries from baseline, from samples that were subjected to a freeze/thaw cycle are shown in Table 3, with further supporting data shown by sample type in Supplemental Tables 1 and 2. A single freeze/thaw cycle was found to be permissible, with sample stability within the set acceptance criteria deviation limits of ≤ 0.100 pg/mL/ ±10% recovery.

**Table 3:** Absolute/relative deviations of plasma pTau181 recoveries after freeze/thaw cycle across different sample types (*n* = 10)

Sample tube	Participant samples*	Mean concentration at t_0_ (pg/mL)	Deviation after a single freeze/thaw cycle	Acceptance criteria	
				Value	Unit
Li-hep	1a 2a 3a 4a 5a 6a 7a 8a 9a 10a	1.87 0.587 1.38 0.753 0.816 0.691 1.93 1.06 0.493 1.00	-2.14% -0.0283 pg/mL -2.82% -0.0267 pg/mL -0.0150 pg/mL -0.00370 pg/mL -0.727% 1.04% -0.0100 pg/mL -3.96%	± 10 ± 0.10 ± 10 ±0.10 ± 0.10 ± 0.10 ± 10 ±10 ± 0.10 ± 10	% pg/mL % pg/mL pg/mL pg/mL % % pg/mL %
K2-EDTA	1b 2b 3b 4b 5b 6b 7b 8b 9b 10b	2.04 0.663 1.51 0.777 0.862 0.766 1.89 1.22 0.481 1.09	-0.294% -0.0159 pg/mL -1.59% -0.0151 pg/mL -0.0125 pg/mL 0.000100 pg/mL 0.0531% -0.493% -0.00200 pg/mL -3.13%	± 10 ± 0.10 ± 10 ±0.10 ± 0.10 ± 0.10 ± 10 ±10 ± 0.10 ± 10	% pg/mL % pg/mL pg/mL pg/mL % % pg/mL %
K3-EDTA	1c 2c 3c 4c 5c 6c 7c 8c 9c 10c	1.99 0.644 1.46 0.758 0.836 0.760 2.01 1.16 0.494 1.06	-0.704% -0.0253 pg/mL -1.99% 0.00350 pg/mL -0.0199 pg/mL -0.00320 pg/mL -1.54% -1.46% -0.00780 pg/mL -2.84%	± 10 ± 0.10 ± 10 ±0.10 ± 0.10 ± 0.10 ± 10 ±10 ± 0.10 ± 10	% pg/mL % pg/mL pg/mL pg/mL % % pg/mL %

*Abbreviations: K2-EDTA* dipotassium ethylenediaminetetraacetic acid, *K3-EDTA* tripotassium ethylenediaminetetraacetic acid, *Li-hep* lithium-heparin, *pTau181* tau phosphorylated at threonine 181, *t*_*0*_ time point 0 (baseline plasma pTau181 measurement)

*Samples a, b, and c were all taken from the same participant

## Discussion

We investigated the effect of sample storage temperatures and duration, as well as a single freeze/thaw cycle, on Elecsys pT181p immunoassay measurements of plasma pTau181 in Li-hep, K2-EDTA, and K3-EDTA tubes. Following optimization of the sample collection and handling procedures, contamination of plasma samples with buffy coat and erythrocytes was prevented. This ensured that the assay results were reliable and met the linear regression acceptance criteria for all storage and handling conditions examined.

The Medical Device Innovation Consortium Specimen Stability Guideline recommends that all short- and long-term stability claims must be supported by testing that extends beyond the intended target claim [30]. Therefore, this study protocol implemented a safety margin of 10–20% and the second-to-last stable time point was designated as the final stability claim. Based on this, our results demonstrate that plasma pTau181 was stable across the tested sample types for a maximum of 2 days at 25 °C, 7 days at 4 °C, 12 weeks at -20 °C, and 16 weeks at -80 °C, and after a single freeze/thaw cycle. This favorable breadth of stability supports the potential utility of the plasma pTau181 biomarker for use in different settings, and importantly, suggests feasibility of its application in frontline healthcare settings such as primary care. Our results suggest that whole blood samples could feasibly be collected in primary care without requirement for complex storage conditions and that the plasma pTau181 analyte would remain stable throughout expected collection, transport, and measurement conditions and timeframes. The recommendation would be for samples to be shipped at 2–8 °C from the collection site to a measurement site, and the resultant plasma can then be stored for up to 2 days at 25 °C (i.e., room temperature), 7 days at 4 °C (i.e., in the refrigerator), or 12 weeks at.

-20 °C (i.e., in the freezer) before measurement. Our finding that plasma samples were also stable for 16 weeks at -80 °C is notable for the application of plasma pTau181 assessment in the clinical trial setting, where this storage temperature is common practice.

There is a current focus on integrating BBBM assays into care pathways for AD, to simplify diagnosis and facilitate access to appropriate treatment, while reducing strain on limited healthcare resources [15, 17, 31–33]. A test that rules out Aβ pathology early in the pathway is of particular interest, as it would help to reduce unnecessary and invasive procedures in individuals with a low likelihood of Aβ pathology, while prioritizing the need for confirmatory testing in those individuals not ruled out [34]. Having established protocols for sample handling and assay use is an important part of the process for implementing these tests into routine care. The stability of the analyte is a key aspect that underscores the feasibility of BBBM testing for AD. Our results confirm the stability of plasma pTau181 during the collection, transport, and measurement processes that would be used in the routine setting. These observations are vital for implementation of this immunoassay, which should help to increase accessibility of early testing for AD.

As BBBM assays become more widely used, it is important that explicit criteria for their application are defined, to obtain the best outcomes for individuals seeking diagnosis and treatment for their cognitive complaints/symptoms. Knowing which individuals to screen is vital, and physicians should consider criteria such as age, risk profile, and early cognitive red flags before initiating any testing procedures. The interpretation of BBBM test results should also be highly standardized and depending on the intended use, a single cutoff with binary classification for the likelihood of Aβ pathology presence (positive and negative) or a dual cutoff approach enabling three-tier classification for the likelihood of Aβ pathology presence (negative, intermediate, and positive) may be appropriate [35]. Furthermore, it is important that it is explicitly stated and understood that a positive BBBM test result does not automatically constitute a diagnosis of AD.

The pT181p immunoassay is intended for use in people aged ≥ 55 years presenting with signs, symptoms, or complaints of cognitive decline [23, 24]. Younger people and those with factors suggesting an alternative underlying cause of their symptoms will need to be appraised using alternative assessments/different diagnostic procedures. In addition, accurate diagnosis in elderly people with multiple comorbidities and mixed pathologies may be challenging, and may require a combination of tests [36]. While implementation of the pT181p immunoassay and early testing in frontline care settings such as primary care appears feasible, training and education will become critical for correct interpretation and sensitive communication, particularly regarding potentially distressing results [36, 37].

The strengths of this study include the breadth of storage conditions, measuring time points, and different sample types investigated to ensure the results are applicable to real-world and clinical trial scenarios. Furthermore, standardized sample collection and processing, together with strict adherence to established biobank quality standards, strengthens the robustness and reliability of the results.

A key learning from this study is that it will be important for other centers and healthcare professionals not to deviate from the recommended collection and handling conditions. Ensuring that the assay protocol and manufacturer instructions are rigorously followed is critical to preventing sample contamination and unmeasurable or misleading results, and to maintain diagnostic confidence within primary care.

### Limitations

The main limitations of this study were the sample size and single-center setting. While a study population of *n* = 10 is typical of short-term stability studies, the resultant data do not preclude potential confounding factors such as age, gender, or comorbidities, which are present in the real-world population. The Elecsys pT181p immunoassay is intended to be used as an aid in the initial assessment for Alzheimer’s disease and other causes of cognitive decline in adults aged 55 years and older, presenting with signs, symptoms, or complaints of cognitive decline. Therefore, to stimulate real-world conditions this study only included samples from the intended-use population (study participants ranged from those with subjective cognitive decline to those with moderate AD dementia), with no individuals outside this population, (such as people without cognitive symptoms), included. Thus, the sample stability results cannot be extrapolated beyond the intended-use population, limiting the generalizability of our findings to broader clinical settings.

In our study, sample stability interpretation relied on a *p* ≥ 0.05 threshold for the linear regression slope. While this significance threshold is widely used, this cutoff may oversimplify the interpretation of the data, as a non-statistically significant deviation from 0 does not demonstrate the complete absence of analyte degradation. Therefore, small changes in the stability of the samples over time cannot be fully excluded in interpretation of these results.

## Conclusions

There is a clear medical need to streamline the AD diagnostic pathway and remove the current barriers to testing to facilitate earlier diagnosis. Implementing a BBBM test to enable early rule out of AD-associated Aβ pathology offers a potential solution. The stability of plasma pTau181 is key for practical implementation of this BBBM test in frontline care settings such as primary care, to increase accessibility of early testing for AD. With correct pre-analytical handling to prevent buffy coat and erythrocyte contamination, we found that plasma pTau181 samples were stable across all measured conditions, and the pT181p immunoassay provided reliable results. These findings may support possible future implementation of the test into primary care, alongside other tools used for early diagnosis of AD. In this context, we have previously developed a structured clinical pathway, with BBBM testing integrated into the workflow, from initial presentation in primary care through biomarker-guided triage to specialist referral. This pathway may serve as a practical framework for implementation and is described in detail elsewhere [38].

## Supplementary Information

Supplementary Material 1.

## Data Availability

The datasets used and/or analyzed during the current study are available from the corresponding author on reasonable request.

## Abbreviations

Aβ: Amyloid-beta
AD: Alzheimer’s disease
BBBM: Blood-based biomarker
CV: Coefficient of variation
K2 EDTA: Dipotassium ethylenediaminetetraacetic acid
K3 EDTA: Tripotassium ethylenediaminetetraacetic acid
Li-hep: Lithium-heparin
pTau181: Tau phosphorylated at threonine 181
pTau181p: Plasma tau phosphorylated at threonine 181
pT181p: Phospho-Tau (181P) Plasma
SD: Standard deviation
t: Time point
tn: Any of the time points 1–5, from one of the designated storage conditions
t0: Time point 0 (baseline plasma pTau181 measurement)
t1–5: Measurement time points 1–5
