# Supplementary material for: A prospective observational study measuring the stability of plasma pTau181 using the Elecsys Phospho-Tau (181P) plasma immunoassay for early detection of Alzheimer’s disease
Source: Alzheimers Res Ther. 2026 Oct 24;18:240. doi: 10.1186/s13195-026-02238-7 (PMC13721401; doi:10.1186/s13195-026-02238-7)
Supplement: Supplementary file 1 [file 13195_2026_2238_MOESM1_ESM.docx]

## Supplementary Materials

**Supplemental Table 1** Descriptive statistics of plasma pTau181 measurements at 25°C
in Li-hep, K2-EDTA, and K3-EDTA (Participant 50)

| **Time point** | ***n*** | **Mean concentration, pg/mL** | **SD** | **CV, %** |
| --- | --- | --- | --- | --- |
| **Li-hep** | | | | |
| t_0_ | 10 | 1.87 | 0.0312 | 1.67 |
| t_1_ | 5 | 1.89 | 0.0277 | 1.47 |
| t_2_ | 5 | 1.87 | 0.0187 | 1.00 |
| t_3_ | 5 | 1.84 | 0.0182 | 0.985 |
| t_4_ | 5 | 1.87 | 0.0114 | 0.611 |
| t_5_ | 5 | 1.90 | 0.0305 | 1.61 |
| **K2-EDTA** | | | | |
| t_0_ | 10 | 2.04 | 0.0212 | 1.04 |
| t_1_ | 5 | 2.03 | 0.0167 | 0.823 |
| t_2_ | 5 | 2.07 | 0.0230 | 1.11 |
| t_3_ | 5 | 2.05 | 0.0311 | 1.52 |
| t_4_ | 5 | 2.03 | 0.0164 | 0.810 |
| t_5_ | 5 | 2.06 | 0.0270 | 1.31 |
| **K3-EDTA** | | | | |
| t_0_ | 10 | 1.99 | 0.0215 | 1.08 |
| t_1_ | 5 | 1.98 | 0.0179 | 0.903 |
| t_2_ | 5 | 2.01 | 0.0187 | 0.931 |
| t_3_ | 5 | 1.99 | 0.0418 | 2.10 |
| t_4_ | 5 | 1.99 | 0.0251 | 1.26 |
| t_5_ | 5 | 2.01 | 0.0130 | 0.648 |

*Abbreviations CV* coefficient of variation, *K2-EDTA* dipotassium ethylenediaminetetraacetic acid, *K3-EDTA* tripotassium ethylenediaminetetraacetic acid, *Li-hep* lithium-heparin,
*pTau181* tau phosphorylated at threonine 181, *SD* standard deviation, *t_0_* time point 0 (baseline plasma pTau181 measurement), *t_1–5_* measurement time points 1–5

**Supplemental Table 2** Descriptive statistics of plasma pTau181 measurements at 25°C
in Li-hep, K2-EDTA, and K3-EDTA (Participant 51)

| **Time point** | ***n*** | **Mean concentration, pg/mL** | | **SD** | | **CV, %** | |  |
| --- | --- | --- | --- | --- | --- | --- | --- | --- |
| **Li-hep** | | | | | | | | |
| t_0_ | 10 | 0.587 | | 0.0139 | | 2.37 | |  |
| t_1_ | 5 | 0.573 | | 0.0189 | | 3.30 | |  |
| t_2_ | 5 | 0.593 | | 0.0110 | | 1.85 | |  |
| t_3_ | 5 | 0.577 | | 0.0220 | | 3.81 | |  |
| t_4_ | 5 | 0.579 | | 0.0139 | | 2.40 | |  |
| t_5_ | 5 | 0.585 | | 0.0199 | | 3.41 | |  |
| **K2-EDTA** | | | | | | | | |
| t_0_ | 10 | 0.663 | 0.0177 | | 2.66 | |  |  |
| t_1_ | 5 | 0.656 | 0.0228 | | 3.48 | |  |  |
| t_2_ | 5 | 0.663 | 0.0159 | | 2.39 | |  |  |
| t_3_ | 5 | 0.662 | 0.0251 | | 3.80 | |  |  |
| t_4_ | 5 | 0.641 | 0.0222 | | 3.47 | |  |  |
| t_5_ | 5 | 0.664 | 0.0200 | | 3.01 | |  |  |
| **K3-EDTA** | | | | | | | | |
| t_0_ | 10 | 0.644 | | 0.0112 | | 1.74 | |  |
| t_1_ | 5 | 0.629 | | 0.0213 | | 3.39 | |  |
| t_2_ | 5 | 0.641 | | 0.0305 | | 4.77 | |  |
| t_3_ | 5 | 0.653 | | 0.0170 | | 2.61 | |  |
| t_4_ | 5 | 0.625 | | 0.0138 | | 2.21 | |  |
| t_5_ | 5 | 0.645 | | 0.00661 | | 1.03 | |  |

*Abbreviations CV* coefficient of variation, *K2-EDTA* dipotassium ethylenediaminetetraacetic acid, *K3-EDTA* tripotassium ethylenediaminetetraacetic acid, *Li-hep* lithium-heparin,
*pTau181* tau phosphorylated at threonine 181, *SD* standard deviation, *t_0_* time point 0 (baseline plasma pTau181 measurement), *t_1–5_* measurement time points 1–5
